# Supplementary material for: Roll-to-plate 0.1-second shear-rolling process at elevated temperature for highly aligned nanopatterns
Source: Nat Commun. 2023 Dec 18;14:8412. doi: 10.1038/s41467-023-43766-2 (PMC10728125; doi:10.1038/s41467-023-43766-2)
Supplement: Supplementary file 1 — Supplementary Information [file 41467_2023_43766_MOESM1_ESM.pdf]

## Supplementary Information

# Roll-to-Plate 0.1-Second Shear-Rolling Process at Elevated Temperature for Highly Aligned Nanopatterns

*Junghyun Cho*[1], *Jinwoo Oh*[1], *Joona Bang*[2], *Jai Hyun Koh*[3], *Hoon Yeub Jeong*[1], *Seungjun Chung*[1,4], *and Jeong Gon Son*[1,4*]

[1]Soft Hybrid Materials Research Center, Korea Institute of Science and Technology (KIST), Seongbuk-gu, Seoul 02792, Republic of Korea

[2]Department of Chemical and Biomolecular Engineering, Korea University, Seongbuk-gu, Seoul 02841, Republic of Korea

[3]Clean Energy Research Center, Korea Institute of Science and Technology (KIST), Seongbuk-gu, Seoul 02792, Republic of Korea

[4]KU-KIST Graduate School of Converging Science and Technology, Korea University, Seongbuk-gu, Seoul 02841, Republic of Korea

*Address correspondence to jgson@kist.re.kr

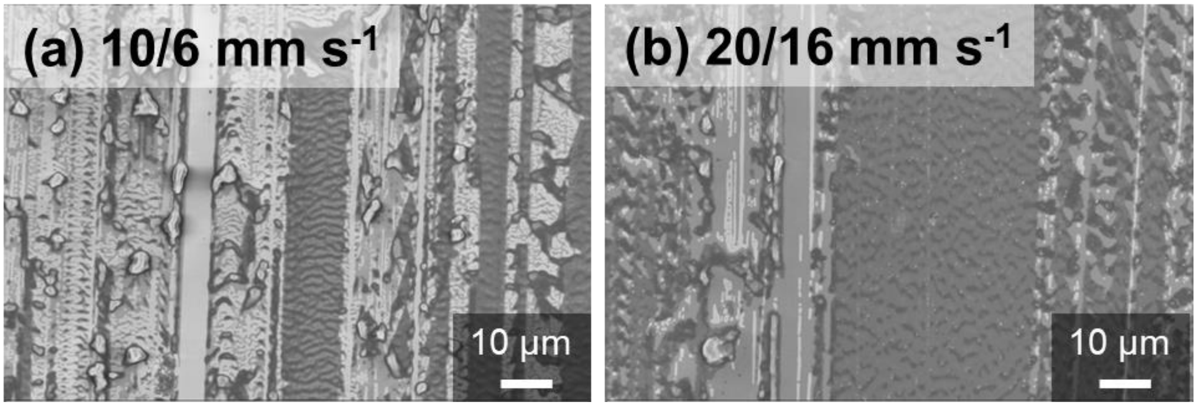


**Figure S1.** SEM images of shear-rolled BCP film with flat PDMS with large ultimate shear strain conditions of (a) 3.6 (10/6 mm $s^{-1}$) and (b) 1.35 (20/16 mm $s^{-1}$).

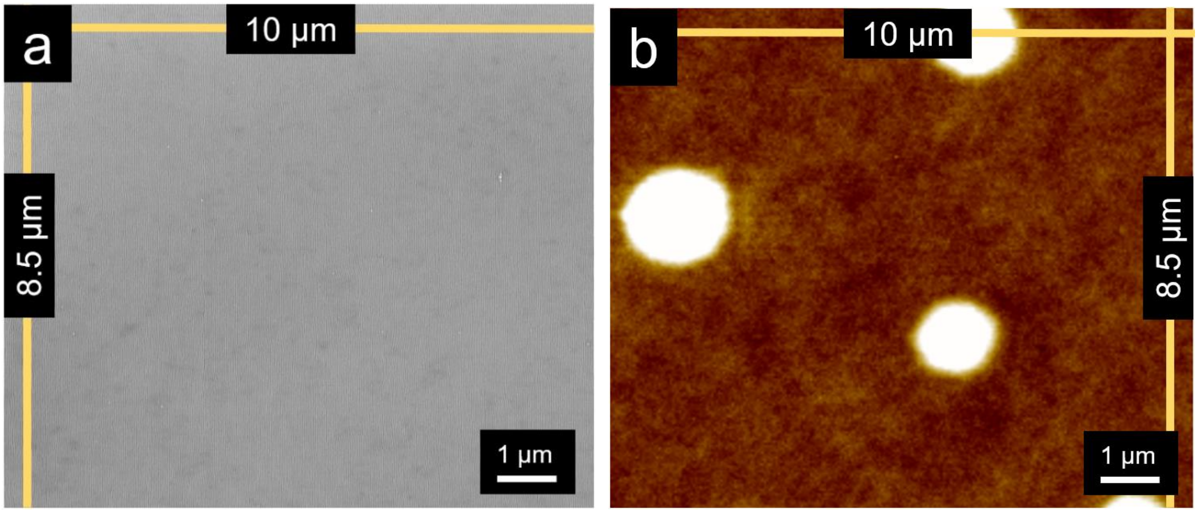


**Figure S2.** Comparison of SEM and AFM data with the same dimensions (10 μm x 8.5 μm). (a) SEM image of shear-rolled BCP film. (b) AFM image of bumpy PDMS. A non-trivial shear field does not form at a bump location.

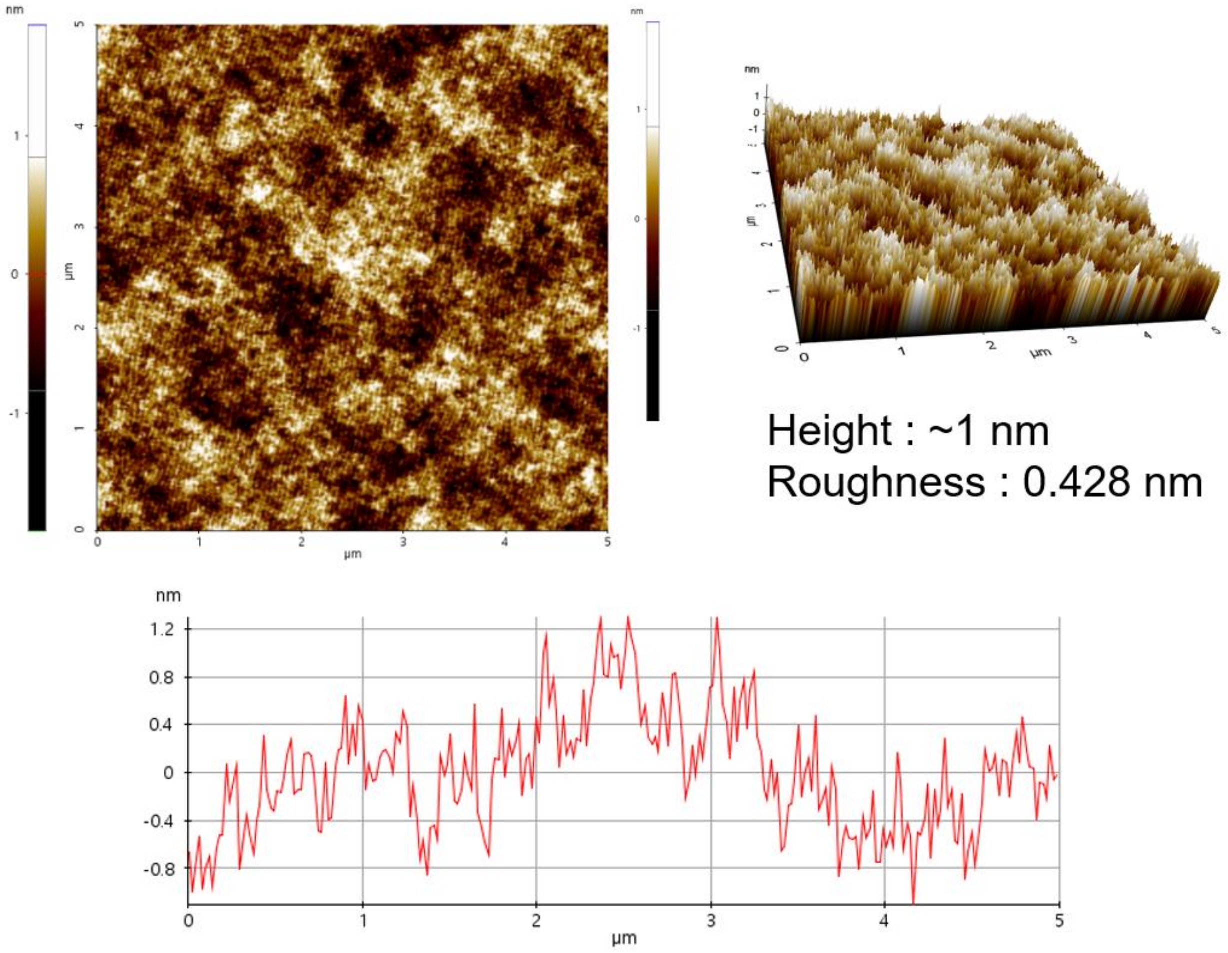


**Figure S3.** AFM results of shear-rolled BCP film surface. (5 µm x 5 µm). The film's surface roughness ($R_q$ = 0.428 nm) of the BCP film after shear-rolling indicates that when bumps apply shear stress to the BCP film, a homogeneous interface is first formed and then the stress is transferred. (height of bump: 25 nm)

**FEM simulation of stress field distribution**

The stress field on the BCP film was calculated using the FEM simulation tool (structural module of COMSOL Multiphysics). The PDMS was modeled as the size of 10 μm x 10 μm x 1 μm rectangular shape. The bump structures were modeled as elliptical shapes with a peak height of 25 nm and a diameter of 2 μm. Four Bump structures were allocated on the PDMS surface with an average distance of 3.5 μm. The BCP film was modeled as the size of 20 μm x 20 μm x 60 nm rectangular shape and was placed with the distance of 500 nm from the PDMS surface. Young's modulus of material was set as 750 kPa for the PDMS and 1 MPa for the BCP film and their Poisson's ratio was set as 0.49 and 0.35. Bumpy surface of PDMS and the top surface of the BCP film were set as contact pair. The bottom surface of the BCP film was designated as the fixed boundary condition. The top surface of the PDMS structure was set as prescribed displacement condition to make a contact with the BCP film. Other boundaries are set as free to allow structural deformation. The contact analysis was performed with the desired contact situation.

In this simulation, we assumed a linear elastic model simply to understand the impact of bumps on the stress field of the BCP film. At high temperatures, the modulus of the BCP film is notably low, allowing it to exist in a mobile state characteristic of the rubbery regime. Therefore, we conducted simulations with the BCP film's modulus minimized to its lowest possible value. The simulations to investigate the stress distribution when normal pressure is applied to the surface of the BCP film with a 10 μm x 10 μm area of PDMS with four bump structures. Based on the trends observed in this normal stress distribution, we predicted the distribution of shear stress. These simulations performed for four different conditions, increasing the pressing depth from 5 nm to 155 nm, each increment being 50 nm. When pressed 5 nm, which is less than the length of the

bump (~20 nm), localized stress field can be observed due to the bump structure (Figure S4a). Even at a pressing depth at 55 nm, the traces of the bumps are clearly visible (Figure S4b). However, as greater pressure is applied to a deeper extent, the localized stress field dissipated into the geometric deformation of PDMS surface which can lead to fully homogenized stress field. In our shear-rolling process, applying pressing depth with 20 μm in the vertical direction leads us to anticipate an even more uniform stress field formation.

To validate our simulation results, we conducted a simple pressing experiment. To minimize the preferential wetting-induced terrace structures and to focus solely on the physical effects caused by the nano-bumps, we executed the PDMS pressing on thicker BCP film (~300 nm) than our experimental conditions (~62 nm). Figure S5 shows the AFM images of pristine PS-b-PMMA films before and after applying pressure with 20 μm of deformation in the vertical direction. In Figure S5a, the pristine PS-b-PMMA film exhibits a relatively flat surface from the spin coating. However, after applying pressing depth with 20 μm at 280 ˚C for 1 s, the film did not leave traces of bumpy structure and rather formed a flatter surface. These results, consistent with simulations, indicate that the BCP films undergo flattening deformation rather than stamping of the bumpy structure when mild pressure is applied, even at high temperatures. Based on these results, it can be concluded that the PDMS pad with nano-bumps can achieve conformal contact and uniform shear transfer to the BCP film if sufficient pressure is applied during the shear-rolling, and can be separated immediately after shear-rolling.

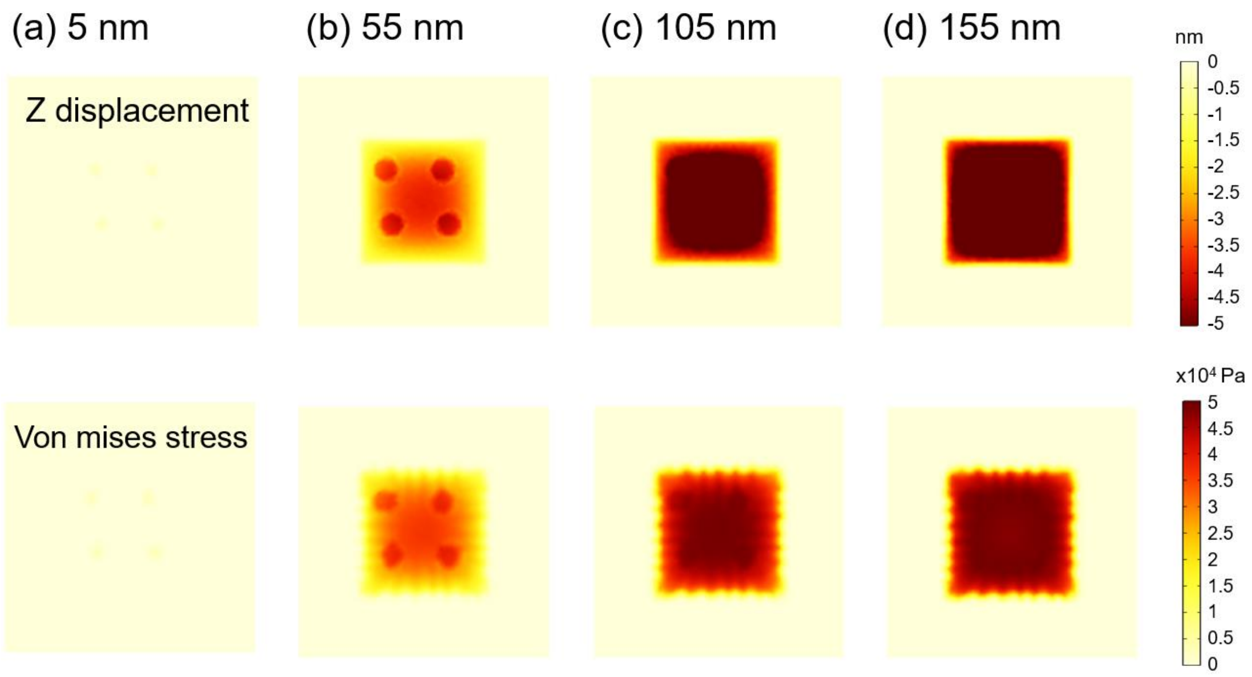


**Figure S4**. FEM simulation results of stress field on the BCP film with different contact displacements. (a) Pressing depth = 5 nm, (b) 55 nm, (c) 105 nm, (d) 155 nm. The unit of z displacement is nm, and Von mises stress is Pa.

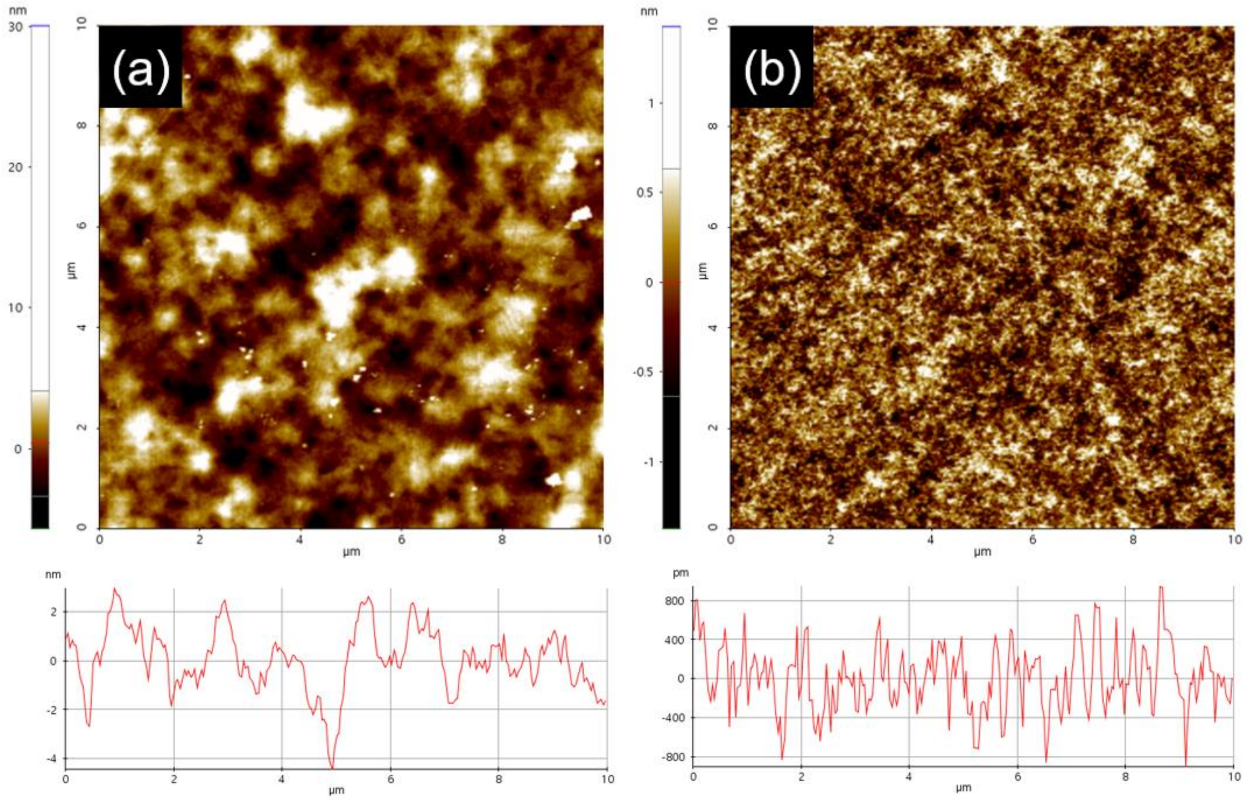

**Figure S5**. AFM results of PS-b-PMMA film surface (a) before and (b) after pressing with 20 μm deformation at 280 ˚C for 1 s.

**The reasons of undulation patterns with flat PDMS and BCP films with bumpy PDMS**

We focus more on in-depth investigation why the bumpy structure led to flat surface and better alignment. Firstly, I would like to tell you about the experimental conditions of shear-rolling we tested in Figure S6. The speed difference between the roller and the substrate was assumed to be 4 mm $s^{-1}$, the moving speed of the substrate was 32 mm $s^{-1}$, and the contact area was measured to be approximately 2.2 mm in Figure S8. And the contact time can be calculated as 0.07 s from contact width and substrate speed. Then, ultimate (end stage) x-axis deformation at the top is around 0.28 mm and ultimate shear strain can be obtained as 0.4 from dividing the PDMS thickness (~0.7 mm), which means that a fairly high deformation occurs. In Figure S1, because we applied a very severe ultimate shear strain over 3 (3 times more lateral deformation than the thickness), we can see the stick-slip mode between the PDMS and BCP films.

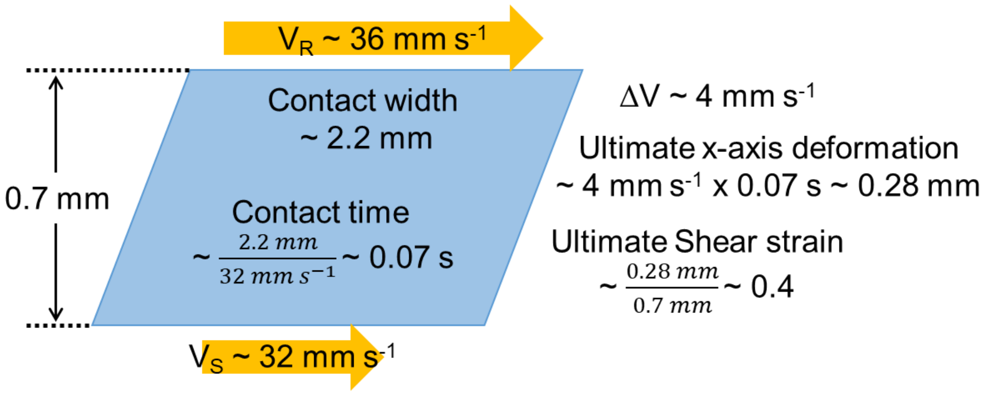


**Figure S6.** Schematic of ultimate deformation of PDMS pad at the last stage in the shear-rolling process.

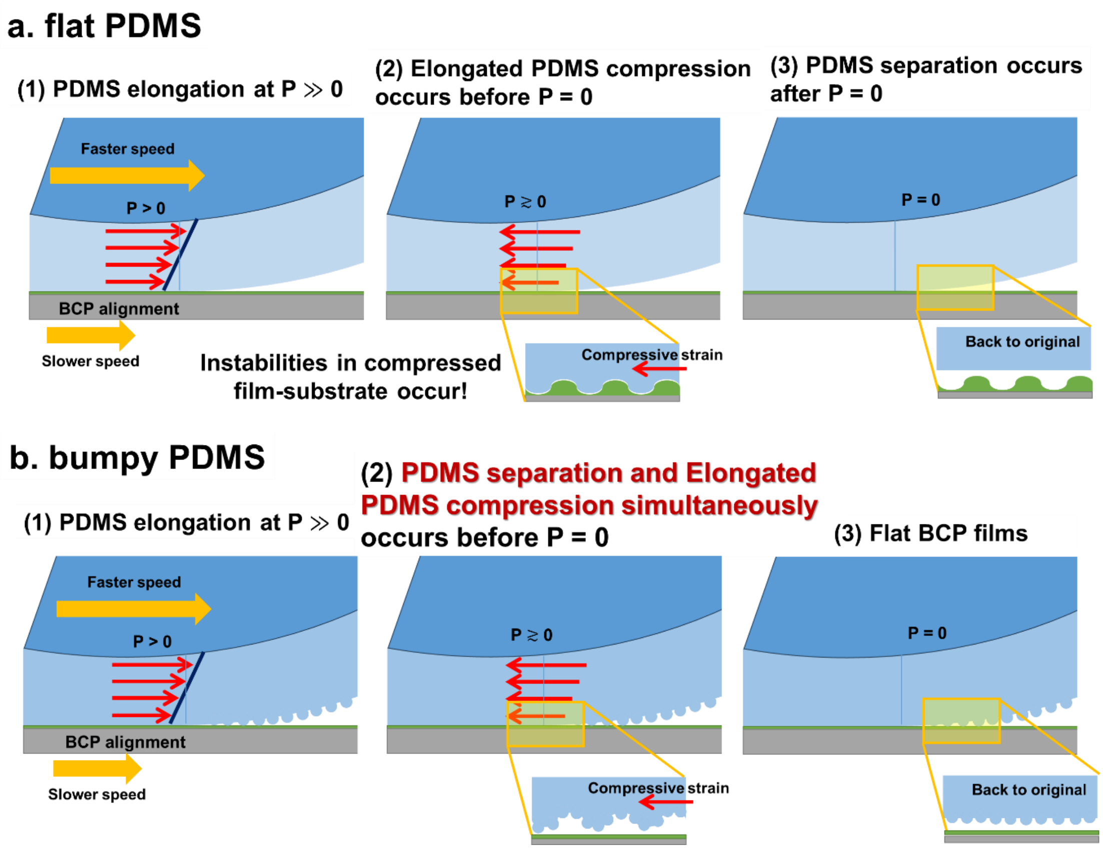


**Figure S7.** Schematics of shear-rolling process at the last stage with (a) flat PDMS and (b) bumpy PDMS.

We now consider the final stage of shear-rolling, as shown in Figure S7. Shear stress is applied to PDMS while the roller and substrate are attached due to shear-rolling with different speed (stage (1)). However, due to the elastic nature of the PDMS, which has already undergone a lot of elongation just before the detachment, the force to bounce back becomes stronger according to Hooke's law. Compressive stress is also transferred to the still attached BCP film due to the compressive strain of the elongated PDMS. Here, we think that instabilities in compressed film-substrate systems, which explain the forming regular period wrinkles when a thin film on a stretchable substrate is subjected to compressive stress, can be applied.[1] According to this hypothesis, before the PDMS and BCP films are separated, that is, just before the pressure becomes 0 (stage (2)), wrinkles can form in the BCP film with different modulus due to compressive

deformation of PDMS.

At this time, the mechanical behaviors of flat PDMS and bumpy PDMS can be different. For flat PDMS, detachment requires a small but additional force (the force from the roller lifting the PDMS) after the pressure becomes zero due to adhesion (Work of adhesion between PDMS and PS/PMMA ~34.5 mN/m at 200 ˚C based on OWRK interfacial tension acquiring method and surface tension data[2]). Therefore, because there is sufficient time for the compressive stress of PDMS to be transferred to the BCP film before detachment (Figure S7a(2)), relatively regular undulations can be formed in the BCP film after the detachment (Figure S7a(3)) due to wrinkle formation at stage 2. On the other hand, for bumpy PDMS, due to the pointed structural nature and elasticity of the bumps, the contact area decreases rapidly and separation also occurs before the pressure reaches 0. In this case, compression of the stretched PDMS and separation can occur simultaneously before the pressure becomes 0 (Figure S7b(2)), thereby avoiding the formation of wrinkles due to compressive strain.

Then, to prove this hypothesis, we checked whether the characteristics of wrinkle formation such as period and amplitude in general compressive strain were also present in this study. The formulas below are the relationship between the critical strain, period, and amplitude of the wrinkles formed by compression of stiff thin films on soft elastic substrates. The key point is that the period is positively correlated with the modulus ratio of the film and is not affected by the applied strain.

wrinkles are stable for large strains

$$\epsilon > \epsilon_c \sim \left(\frac{E_s}{E_m}\right)^{2/3}$$

wavelength of wrinkles

$$\lambda \sim d\left(\frac{E_m}{E_s}\right)^{1/3}$$

amplitude of wrinkles

$$h_0 = \frac{\lambda}{\pi}\sqrt{\frac{\Delta}{L}} = \frac{\lambda\sqrt{\epsilon}}{\pi}$$

Now, we reinvestigate the results in Figure 3. Increasing the pressing height from the roller, undulation patterns are clearly visible. The period does not change significantly at approximately 10 μm. Additionally, the undulation becomes more evident depending on the shear rate. As calculated above, the contact area increases with the pressing height and the contact time also increases. The ultimate shear strain increases accordingly. Additionally, as the shear rate increases, the ultimate shear strain also increases at the same period of time. Because the degree (and ultimately amplitude) of wrinkles increases as the ultimate shear strain increases but the period does not change, we confirm that our undulation patterns can originate from the wrinkle formation due to compression of the bilayer film. Figure 4 (with bumpy PDMS) also shows that the contact time is reduced by gradually increasing the speed of the substrate at the same shear rate. Reducing the contact time also reduces the ultimate shear strain. As a result, the amplitude of the wrinkles formed is reduced, reducing their impact, and a flat BCP film can be obtained. As the speed of the substrate increases, the undulation diminishes and there is no significant difference in the period, which can be judged to be caused by the wrinkling phenomenon from compressive deformation rather than the large velocity-originated slip-stick phenomenon.

**Calculation of contact width and maximum shear strain on PDMS**

Contact width and Maximum shear strain can be calculated from the height at which maximum pressure is applied. The figure on the right indicates the geometry at the point of contact between the roller and PDMS. **w** denotes the width at the maximum contact

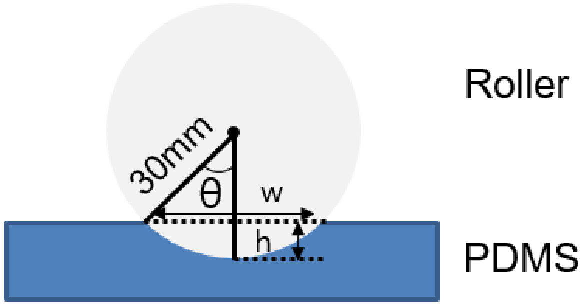

of the PDMS. **h** is an empirical parameter that changes to the height adjustment device and denotes PDMS deformation height. The width can get from the height value. Calculations are below,

$$\cos\theta = \frac{30-h}{30}, \quad \mathrm{w} = 2 \cdot 30 \cdot \sin\theta$$

and we can consider the rolling time **t** as the distance width passes.

$$\mathrm{t} = \frac{\mathrm{w}}{v_{stage}}$$

Shear displacement D is,

$$\mathrm{D} = \mathrm{t} \cdot \Delta\mathrm{v}$$

Finally, the maximum shear strain is calculated as follows.

$$\gamma_{max} = \frac{D}{H(Thickness\ of\ PDMS)}$$

The uniformity of the film was evaluated by the maximum shear strain obtained from each value through controlling the empirical parameters.

In addition, the distance between the roller and the BCP surface, known as the pressing height, was calculated and compared to experimental values of w, the width at the maximum contact of the PDMS in order to gain an accurate value of the height.

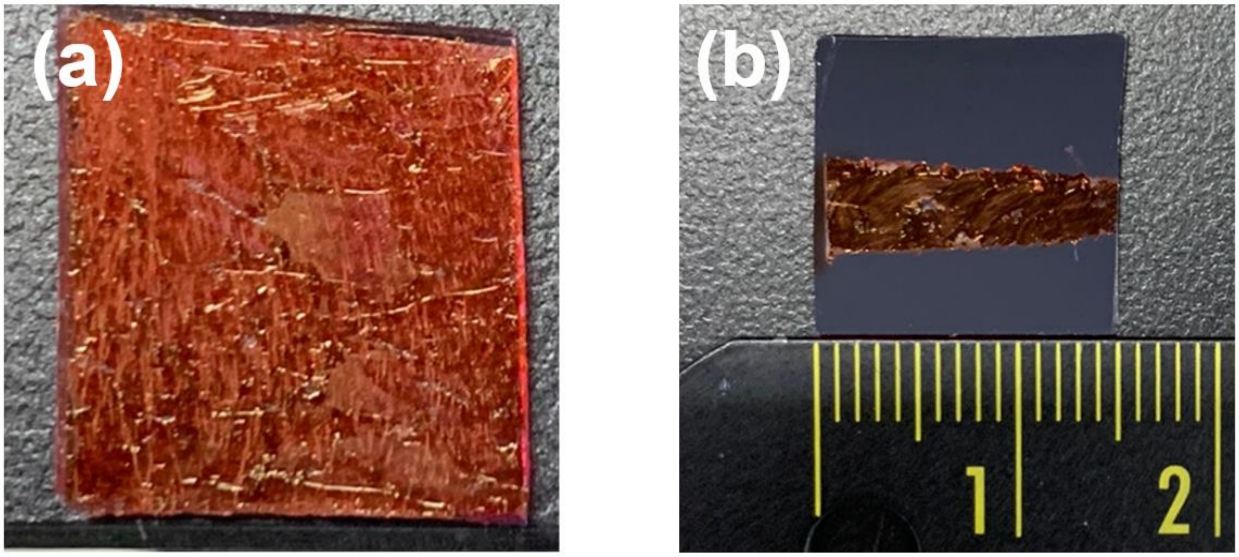


**Figure S8**. Measurement of contact width at the moment of contact by an ink stamping method. (a) An image with ink coating the entire surface of the PDMS. (b) Image of transferred ink at the moment of contact at which maximum pressure is applied. The ruler denotes the dimension of the Si wafer and PDMS.

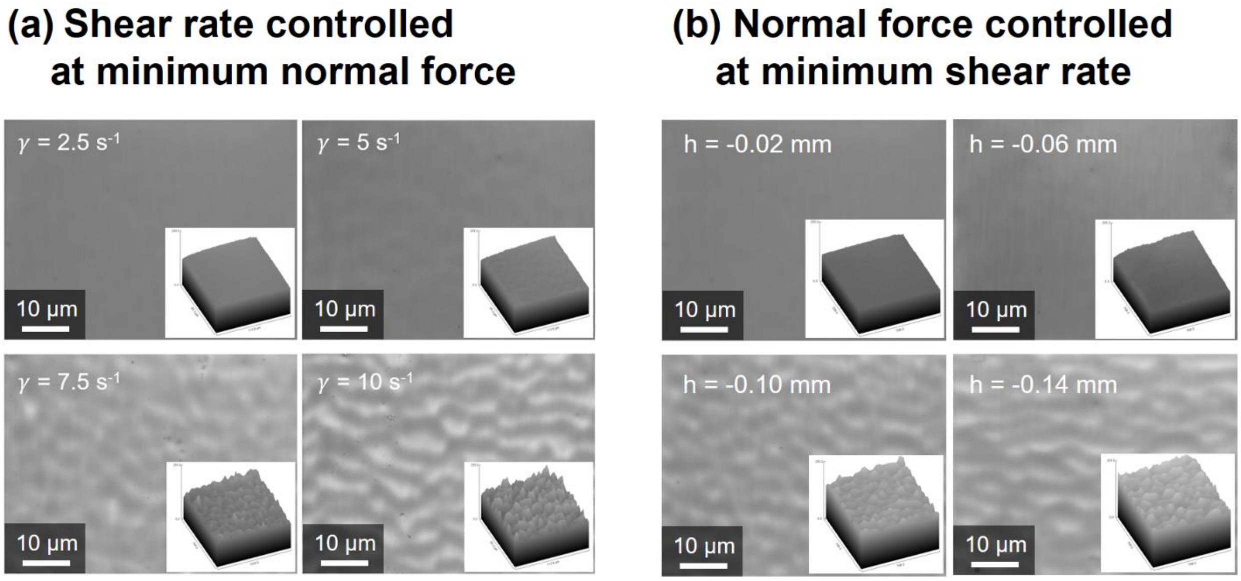


**Figure S9**. Low magnification SEM images of film surface instability with changes in shear rate and normal force. (a) four different shear rates controlled at minimum height (h = 0.02 mm). (b) four different normal forces controlled at the minimum shear rate ($\Delta v$ = 2 mm/s). Insets are surface plots from ImageJ.

**Determine the orientation order from Herman's orientation parameter**

The degree of orientation was quantified with Herman's orientation parameter as defined in following equation.[3,4]

$$f_{2D} = 2 < \cos^2\theta >_{2D} - 1$$

and

$$< cos^2\theta >_{2D} = \frac{\int_0^{\pi} I(\theta)_{2D} cos^2\theta d\theta}{\int_0^{\pi} I(\theta)_{2D} d\theta}$$

,where θ is the local orientation angle and I(θ) is the intensity or popularity at θ. The Herman's orientation parameter takes the value 1 for a perfect orientation parallel to the director and transversely aligned value is -0.5. Non-oriented cases have an 0 value.

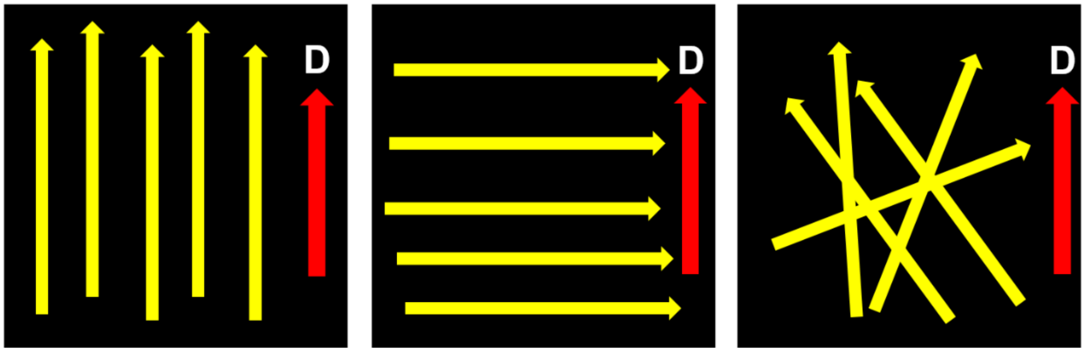


f = 1 f = -0.5 f = 0

**Figure S10**. Values for the Herman's orientation parameter for three cases. Perfect aligned (f = 1), transversely aligned (f = -1), random oriented (f = 0), respectively.

Additionally, for the quantitative analysis of orientation order and visualizing our nanopattern using color, we conducted orientation analysis in ImageJ. The structure tensor is derived from Cubic spline gradient of the image and provides information about the local image gradients in different directions. During this process, we were able to set the local window of the region of interest (ROI) in pixel units. Considering our goal of visualizing defects such as dislocations, we

set the minimum window size to 2 pixels, which allows us to distinguish line patterns in one direction while ensuring that defect nodes with an average length of 16 nm (about 7.2 pixels) are color-coded accordingly. In Figure S11, we can observe whether there is a difference in color distinction for dislocation defects.

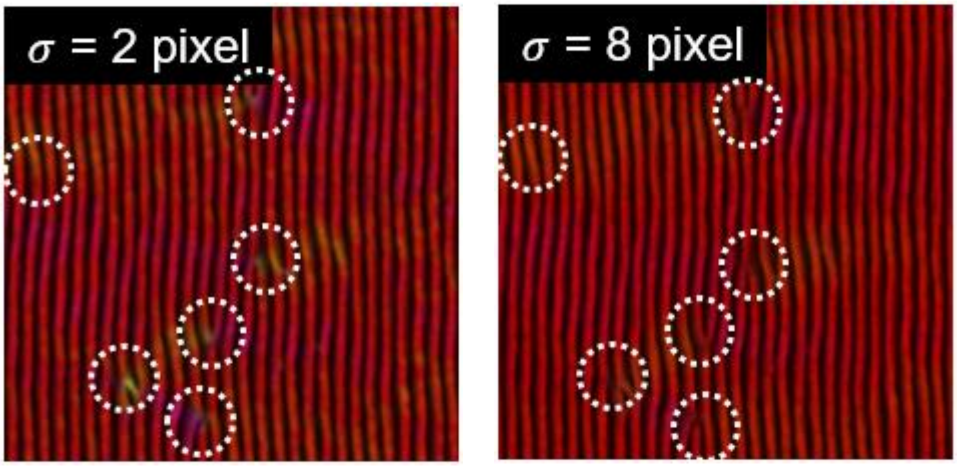


**Figure S11**. Effect of color local window size selection on color differentiation.

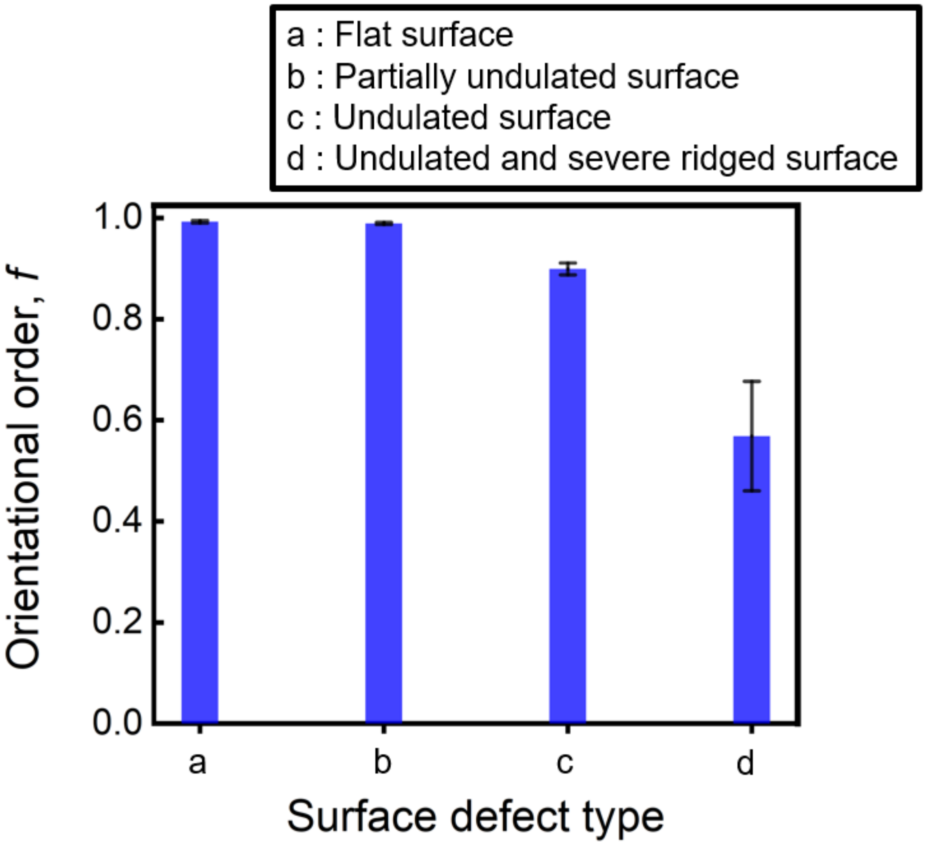


**Figure S12**. Orientation order graphs according to the surface defect type. (a) Flat, (b) Partially undulated, (c) Undulated, (d) Undulated and severe ridged surface, respectively. Error bar represent the standard error of measured orientational order from at least five independent experiments. Source data are provided as a Source Data file.

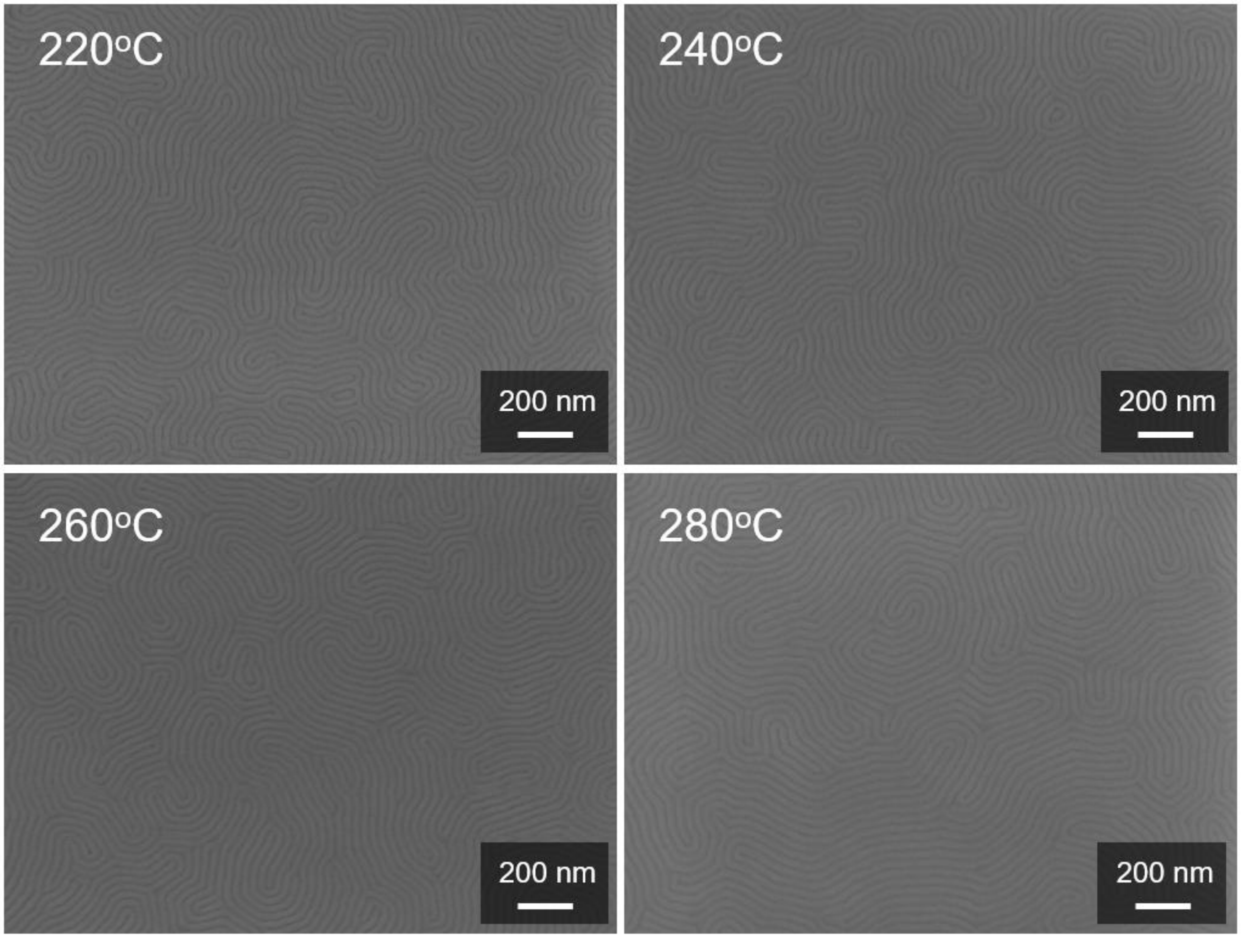


**Figure S13.** SEM images of PS-*b*-PMMA 1 min. pre-thermal annealing on stage before applying shear stress at each temperature. The perpendicular orientation structures were achieved with a short annealing time and without any surface treatment.

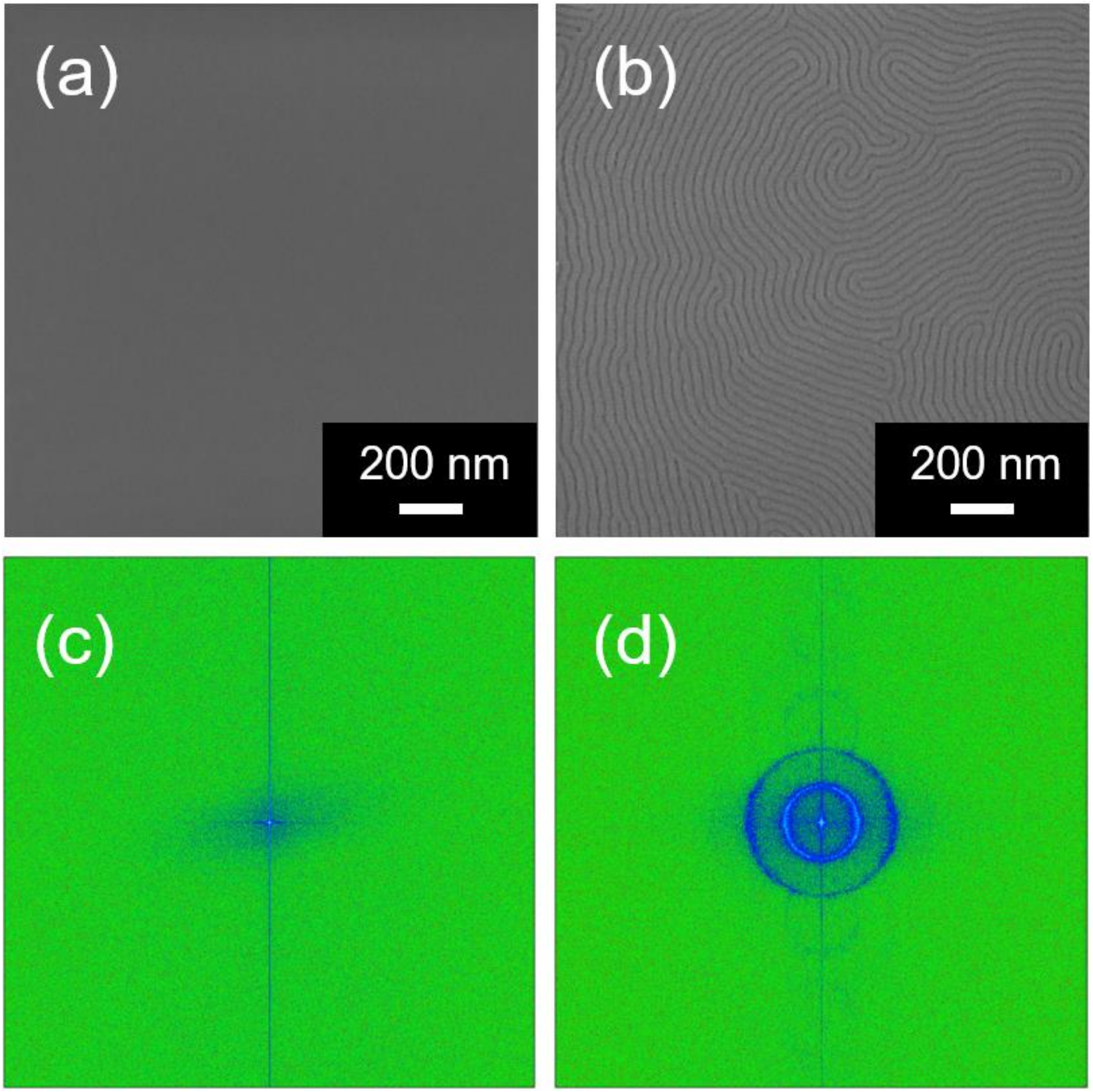


**Figure S14**. SEM images of thermal annealing w/ and w/o PDMS on the top surface of BCP film. (a) The PS-*b*-PMMA film in contact with the PDMS pad for only first 2 seconds of the thermal annealing at 280 ºC formed a parallel orientation from the top surface. (b) The perpendicular oriented structure of PS-*b*-PMMA after thermal annealing at 280 °C. The thermal annealing time is equal to 1 min. for both (a) and (b). Images (c) and (d) respectively show the FFT results of (a) and (b).

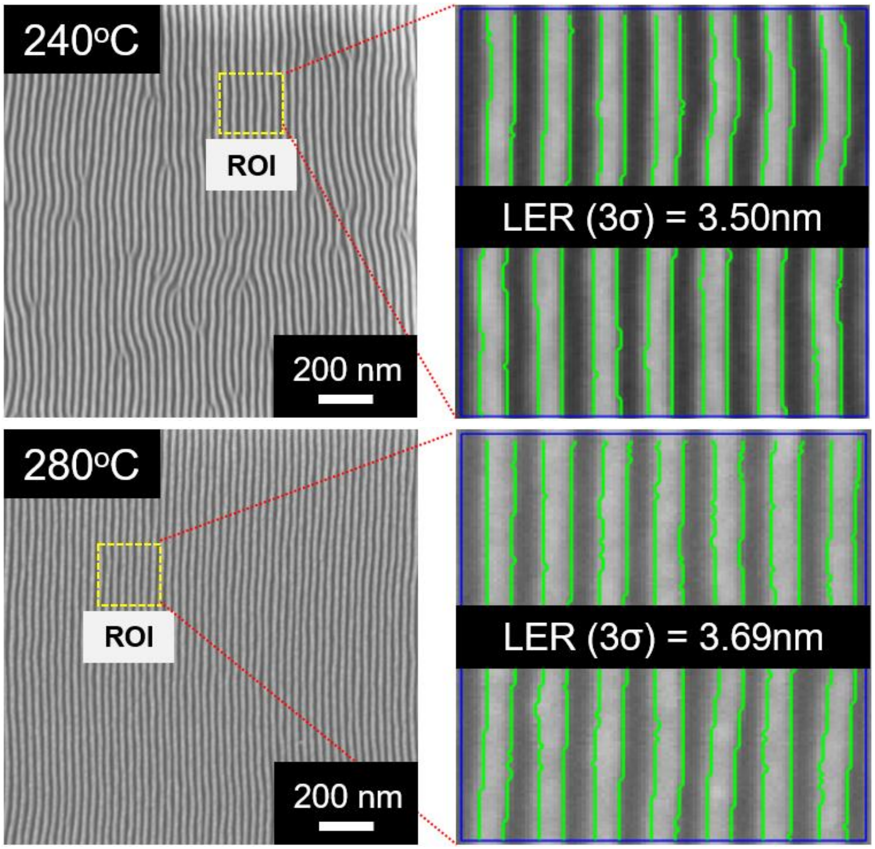


**Figure S15.** Comparison of Line-edge roughness (LER) values at 240 °C and 280 °C. LER is defined as 3 times its standard deviation σ. The LER data of line-space nanopattern was obtained by selecting a region of interest (ROI) then calculated using Lacerm (Line and Contact Edge Roughness Meter) program written in Matlab code. This program was developed by Dr. Cong Que Dinh and is available for use at www.lacerm.com.

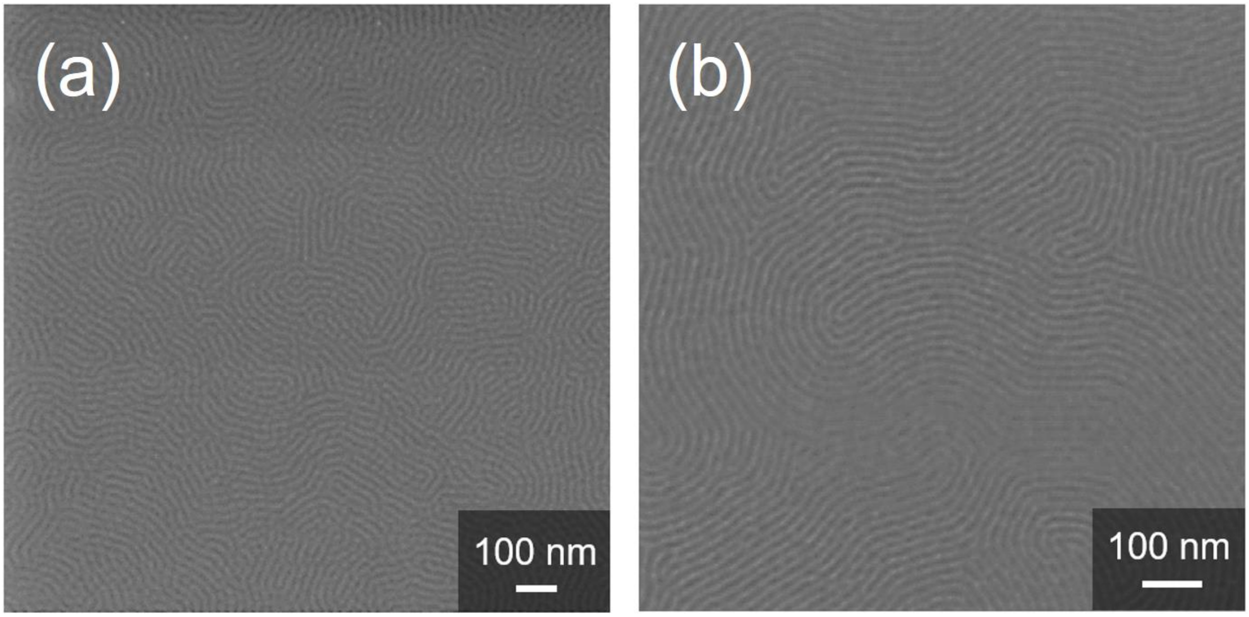


**Figure S16**. SEM images of the perpendicular orientation at a stable temperature condition of topcoat. (a) P2VP-*b*-PS-*b*-P2VP film at 245 ºC. (b) PTMSS-*b*-PMOST film at 245 ºC. The thermal annealing time is equal to 1 min, and $O_2$ RIE (90 W, 20 sccm, 10 sec) was executed for intensifying contrast in the SEM observation for both (a) and (b).

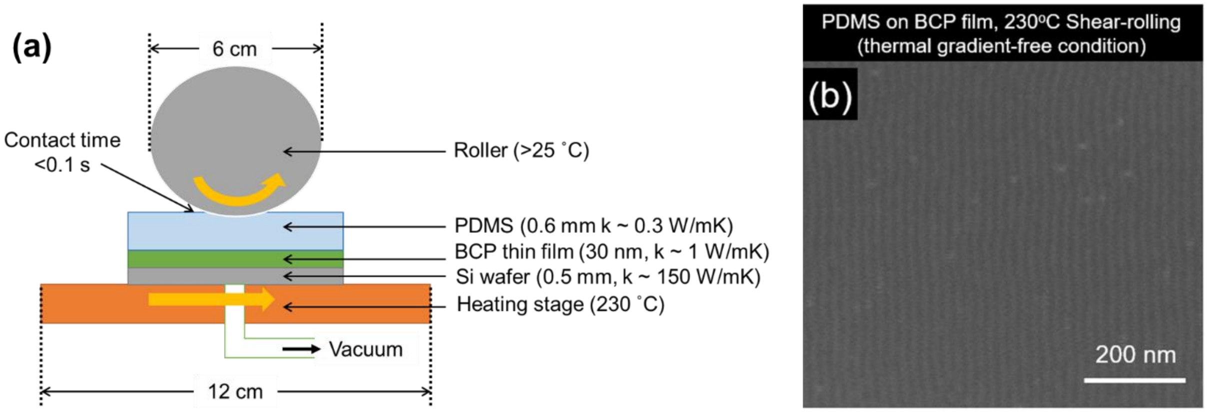


**Figure S17.** (a) A scheme for the shear-rolling process with pre-contact with PDMS pad and pre-annealing for 5 min before the shear. (b) SEM image of PDMS pad pre-contacted P2VP-*b*-PS-*b*-P2VP films after 5 min pre-annealing and subsequent shear-rolling at 230 ˚C (thermal equilibrium condition).

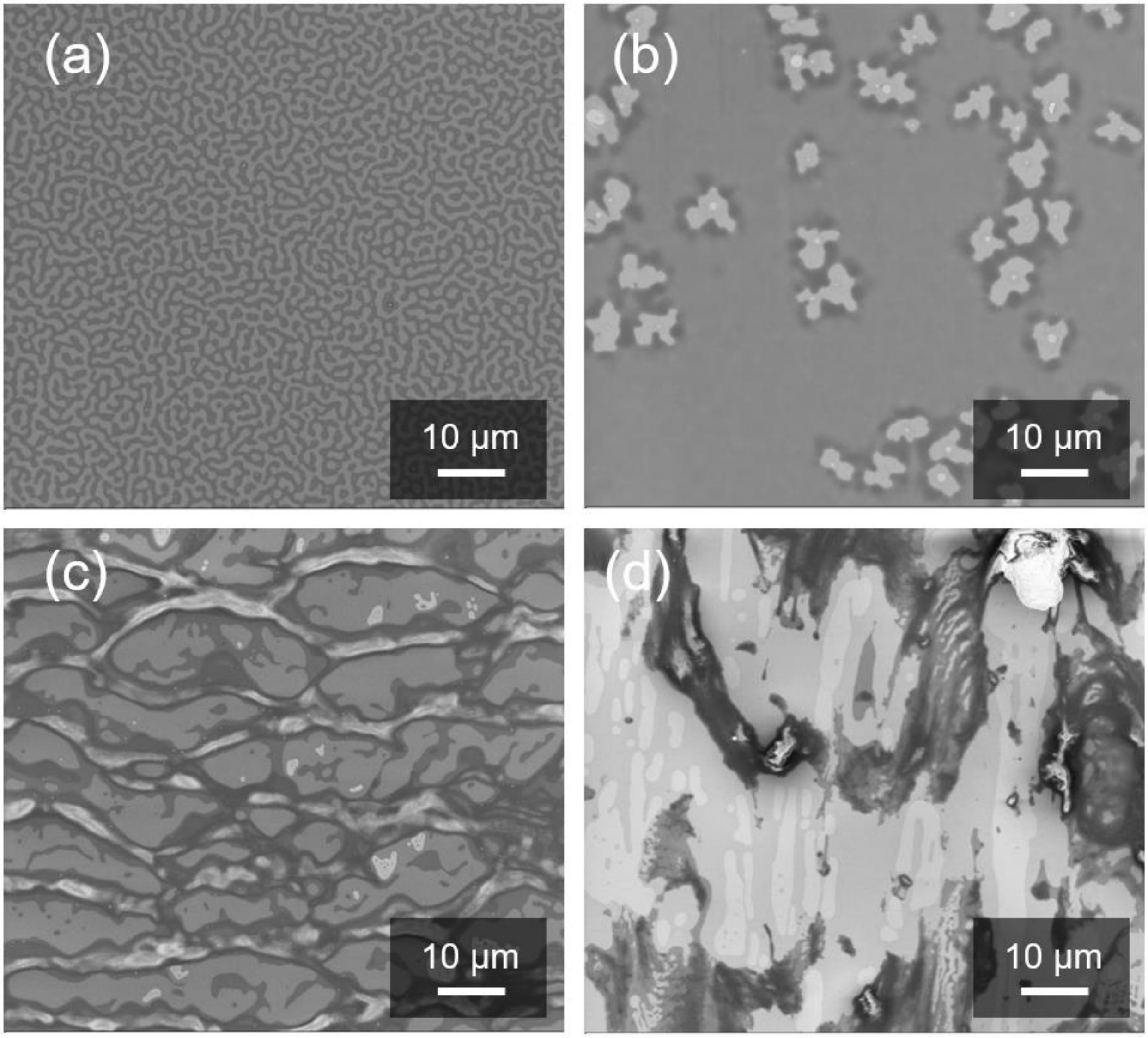


**Figure S18.** Images of instability of the film surface caused by an unstable top coat at high annealing temperature, as well as the undulated film generated by the shear-rolling process. (a) An unstable neutral layer of P2VP-*b*-PS-*b*-P2VP at 240 ºC. (b) Dewetted top coat at 250 ºC 1 min. pre-thermal annealing of PTMSS-*b*-PMOST film. (c) After shear-rolling the P2VP-*b*-PS-*b*-P2VP film at a temperature exceeding 245 °C, and (d) After shear-rolling the PTMSS-*b*-PMOST film at a temperature exceeding 245 °C.

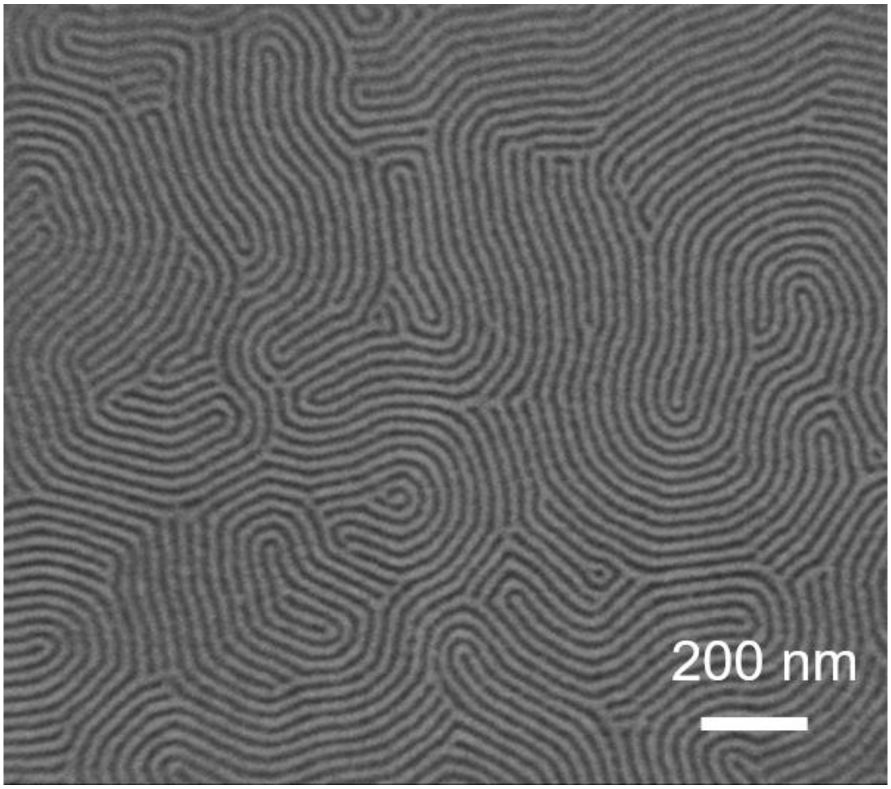


**Figure S19.** PS-*b*-PMMA on polyimide film after 280 ºC 1 min. thermal annealing.

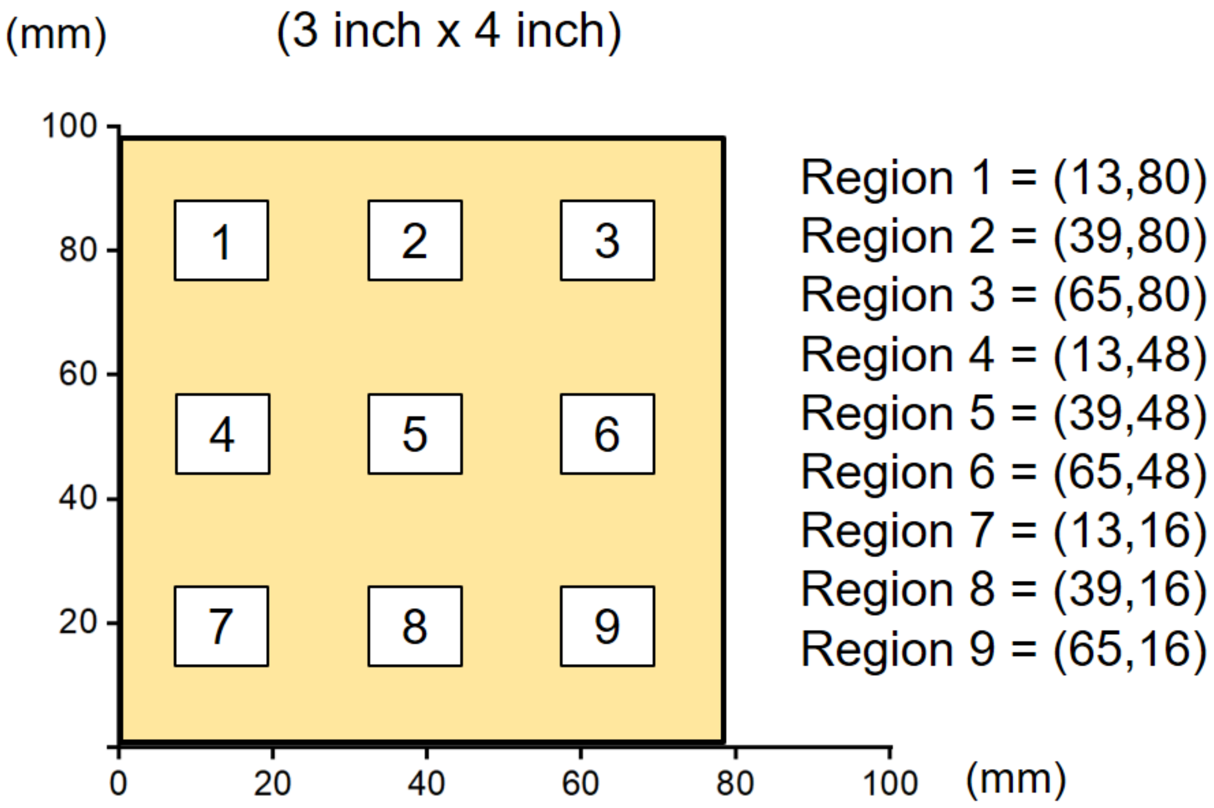


**Figure S20.** The 3-inch x 4-inch nano-patterned PS-*b*-PMMA film on polyimide substrate, which was divided into 9 regions, and the coordinates corresponding to the center of each section are indicated in the figure.

## Reference


1. Wang, Q. & Zhao, X. A three-dimensional phase diagram of growth-induced surface instabilities. *Sci Rep* **5**, 8887 (2015).
2. Wu, S. Surface and Interfacial Tensions of Polymers, Oligomers, Plasticizers, and Organic Pigments. in *Polymer Handbook* (eds. Brandrup, J., Immergut, E. H., Grulke, E. A., Abe, A. & Bloch, D. R.) 521–541 (John Wiley & Sons, 1999).
3. Murphy, J. N., Harris, K. D. & Buriak, J. M. Automated defect and correlation length analysis of block copolymer thin film nanopatterns. *PLoS One* **10**, e0133088 (2015).
4. Kaniyoor, A., Gspann, T. S., Mizen, J. E. & Elliott, J. A. Quantifying alignment in carbon nanotube yarns and similar two-dimensional anisotropic systems. *J Appl Polym Sci* **138**, e50939 (2021).
