# Supplementary figures and images for: Roll-to-plate 0.1-second shear-rolling process at elevated temperature for highly aligned nanopatterns
Source: Nat Commun. 2023 Dec 18;14:8412. doi: 10.1038/s41467-023-43766-2 (PMC10728125; doi:10.1038/s41467-023-43766-2)

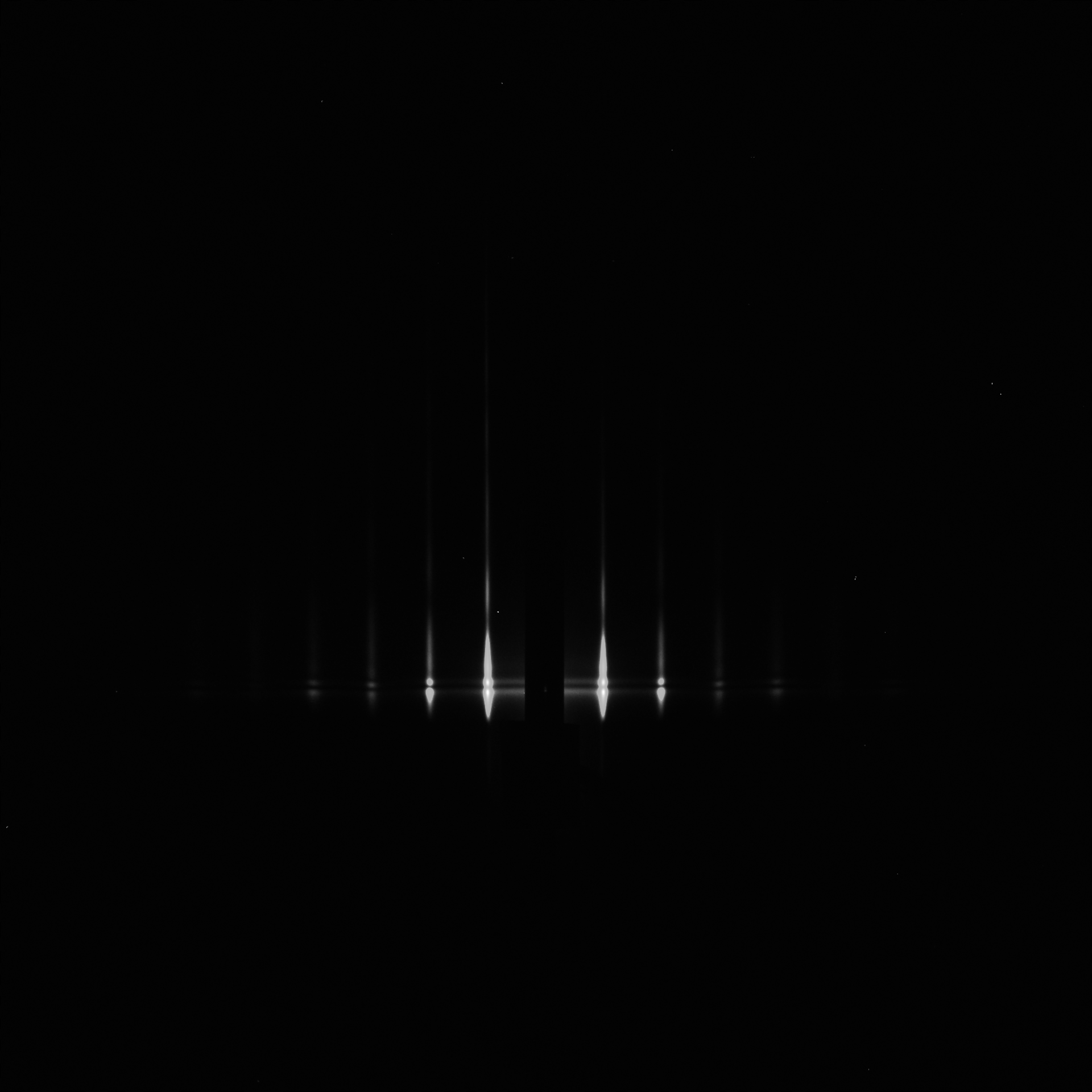

Supplement: Supplementary file 3 — Source Data [file 41467_2023_43766_MOESM3_ESM.zip › GISAXS data/HS-2.5mGI_S07_att3.3335_0.12deg_10sec_0023.tif]
